# Cross-Linked Sulfonated Poly(arylene ether sulfone) Membrane Using Polymeric Cross-Linkers for Polymer Electrolyte Membrane Fuel Cell Applications

**DOI:** 10.3390/membranes13010007

**Published:** 2022-12-21

**Authors:** Junghwan Kim, Seansoo Hwang, Yu-Gyeong Jeong, Yong-Seok Choi, Kihyun Kim

**Affiliations:** 1 Center for Hydrogen·Fuel Cell Research, Korea Institute of Science and Technology (KIST), Hwarang-ro 14-gil 5, Seongbuk-gu, Seoul 02792, Republic of Korea; 2 Department of Materials Engineering and Convergence Technology, Gyeongsang National University, Jinju 52828, Republic of Korea; 3 Composites Materials Application Research Center, Korea Institute of Science and Technology, 92 Chudong-ro, Bongdong-eup, Wanju-gun, Jeonbuk 55324, Republic of Korea

**Keywords:** polymer electrolyte membrane fuel cell, polymer electrolyte membrane, sulfonated poly(arylene ether sulfone), polymeric cross-linker, cross-linked membrane, hydrophilic/hydrophobic, high degree of sulfonation, proton conductivity, physicochemical property

## Abstract

Cross-linked membranes for polymer electrolyte membrane fuel cell application are prepared using highly sulfonated poly(arylene ether sulfone) (SPAES) and polymeric cross-linkers having different hydrophilicities by facile in-situ casting and heating processes. From the advantage of the cross-linked structures made with the use of polymeric cross-linkers, a stable membrane can be obtained even though the polymer matrix with a very high degree of sulfonation was used. In particular, hydrophilic cross-linker is found to be effective in improving physicochemical properties of the cross-linked membranes and at the same time showing reasonable proton conductivity. Accordingly, membrane electrode assembly made from the cross-linked membrane prepared by using hydrophilic polymeric cross-linker exhibits outstanding cell performance under high temperature and low relative humidity conditions (e.g., maximum power density of 176.4 mW cm^−2^ at 120 °C and 40% RH).

## 1. Introduction

In anticipation of a hydrogen society to cope with the current global climate issue, research on the utilization of renewable energy is being actively conducted. However, renewable energy sources are unstable and intermittent during generation, and thus these valuable electrical energies are difficult to apply continuously and stably [1]. Green hydrogen can be prepared from renewable energy and then used for power generation by fuel cells to suppress the peak fluctuations and realize the functions of peak regulations for renewable energy sources. Therefore, related technologies such as fuel cells, electrolysis, and hydrogen storage systems are being widely investigated. Electrical energy production through fuel cell technology has received considerable attention due to its environmental benignity [2]. Polymer electrolyte membrane fuel cells (PEMFCs) are the most explored fuel cell systems due to their high energy conversion efficiency compared to other fuel cell systems and their fast start-up [3,4]. During the PEMFC operation, electrochemical reactions such as hydrogen oxidation reaction (HOR) and oxygen reduction reaction (ORR) occur at anode and cathode, respectively, producing electrical energy. Proton (H^+^) released from the oxidation of hydrogen fed at the anode side is transferred to the cathode side through the electrolyte to react with oxygen [5]. In this type, a polymer electrolyte membrane (PEM) is used as a conducting medium that facilitates proton transfer and as a separator that divides the anode and cathode. To achieve an efficient PEMFC system, PEMs with high proton conducting ability, good physicochemical stability, excellent durability, superior water retention properties, high resistance to fuel crossover, and excellent antioxidant stability in practical fuel cell operations must be developed [5,6].

Currently, perfluorosulfonic acid (PFSA) ionomers have been widely commercialized and used as PEM materials [7]. The structure of PFSA ionomers has a hydrophobic backbone and hydrophilic side chains having sulfonic acid groups at their end group. When PFSA is humidified, the sulfonic acid groups can absorb water and dissociate to form mobile protons, while the microchannel generated by the hydrophilic/hydrophobic phase separation provides a proton transfer pathway [8]. However, because of their high production costs from complex fluorine chemistry during preparation, low operating temperatures originating from their low glass transition temperature (<80 °C), and weaknesses in durability, alternative PEM materials are sought [9,10]. In particular, sulfonated hydrocarbon polymers (SHPs), such as sulfonated poly(arylene ether sulfone) (SPAES) [11,12,13,14], sulfonated poly(ether ether ketone) (SPEEK) [15,16], and sulfonated poly(phenylene oxide) (SPPO) [17,18], are regarded as promising alternative materials because of their high structure designability, excellent thermomechanical properties, and good cost efficiency. The properties of these SHP-based membranes are considerably affected by the chemical structure of the ionomers and their degree of sulfonation (DS) [6]. Typically, SHP membranes with high DS exhibit high proton conductivity, but the physicochemical properties of the membrane show poor long-term performance [19,20]. Therefore, different strategies have been employed to prepare highly conductive and mechanically stable membranes. Instruction of cross-linked structure is known as an efficient method to enhance the mechanical robustness of the membrane, as compact molecular structures through inter-chain bonds contribute to the prevention of deformation that normally occurs due to the dissolution or excessive swelling of the hydrophilic polymer membranes [21]. However, the mobility of the interconnected polymer chains is seriously reduced [16,22]. Additionally, the sulfonic acid groups are sometimes consumed to form the cross-linked structures. Consequently, these reduce the proton conductivity of the cross-linked membranes. Therefore, designing an effective method to introduce cross-linked structures while maintaining the proton conductivity of the membrane is beneficial for the development of high-performance SHP-based PEMs.

In this study, we prepared cross-linked membranes using a SPAES with DS of 70 mol% and polymeric cross-linkers. Since linear SPAES membrane cannot maintain its shape stably under high temperature and wet conditions, cross-linked structure should be induced to enhance the membrane stability. It was reported that polymeric cross-linkers can lessen the degree of chain mobility reduction originated by the formation of cross-linked structure, thereby producing structures with good physicochemical properties and reasonable proton conductivity [23,24]. Two polymers with different DS values were synthesized to systematically investigate the effect of the hydrophilicity of the cross-linkers on the physical and electrochemical properties of the membranes. A detailed description of the preparation and analysis of cross-linked membranes with different cross-linkers is discussed in this paper. Furthermore, preliminary fuel cell performance using the cross-linked membrane was conducted at high-temperature and low RH (120 °C and 40% RH) conditions to investigate the feasibility of the cross-linked membrane as a PEM for high-temperature PEMFCs.

## 2. Experimental

### 2.1. Materials

The information regarding all solvents and reagents used in this study is described in the Supplementary Materials.

### 2.2. Synthesis of Sulfonated Poly(arylene ether sulfone) (SPAES)

SPAES was synthesized through a condensation polymerization reaction of the dihydroxy monomer (4,4′-dihydroxybiphenyl (BP)) with a mixture of dichloro monomers (3,3′-disulfonated-4,4′-dichlorodiphenylsulfone (SDCDPS) and 4,4′-dichlorodiphenyl sulfone (DCDPS)), as described in our previous report (Figure S1) [25]. The SDCDPS/DCDPS feed molar ratio was 70:30. After drying in a 60 °C vacuum oven for 12 h, off-white SPAES was obtained at a yield of 87%. SPAES: proton nuclear magnetic resonance (^1^H-NMR) (deuterated dimethyl sulfoxide (DMSO-*d_6_*), 400 MHz): δ 8.31 (br, 2H, ArH), 7.96 (br, 4H, ArH), 7.87 (br, 2H, ArH), 7.73 (br, 8H, ArH), 7.21 (br, 8H, ArH), 7.12 (br, 4H, ArH), 7.03 (br, 2H, ArH).

### 2.3. Modification of SPAES to SPAES with Chloromethyl Moieties (SPAES-Cl)

SPAES was modified to SPAES-Cl through a substitution reaction using chloromethyl methyl ether and SnCl_4_ as the reagent and catalyst (Figure S1), respectively [24]. A solution containing 7.984 g (15.10 mmol of repeat units) of SPAES in 133.1 mL of *N,N*-dimethylacetamide (DMAc) was added into a 250 mL two-neck, round-bottom flask equipped with a condenser and a magnetic stirrer maintained under N_2_ atmosphere. Then, 4.138 g (15.10 mmol) of SnCl_4_ and 1.939 g (22.63 mmol) of chloromethyl methyl ether were added to the reactor at 25 °C. The solution mixture was heated at 50 °C and stirred for 24 h. The mixture was then poured into excess isopropyl alcohol (IPA). The precipitate was filtered and rinsed several times with methanol and deionized (DI) water. After drying at 60 °C in a vacuum oven for 12 h, SPAES-Cl was obtained (yield = 85%). As confirmed through ^1^H-NMR, the concentration of the chloromethyl groups in SPAES-Cl was approximately 6 mol%, indicating that 0.06 equivalents of the chloromethyl groups were introduced into the repeating unit.

### 2.4. Synthesis of Thiophenoxide-Terminated Polymeric Cross-Linkers

Polymeric cross-linkers with different hydrophilicity (DS = 0, 100 mol%) were synthesized through the condensation polymerization of dithiol monomers and dichloro monomers (Figure S2). Thiophenoxide-terminated sulfonated poly(arylene thioether sulfone) (SDT) was prepared through a condensation polymerization reaction using 4,4′-thiobisbenzenethiol (TBBT) and SDCDPS. Polymerization was carried out in a 250 mL two-neck, round-bottom flask with a Dean–Stark trap, condenser, N_2_ gas inlet and outlet, and stirring bar. Then, a mixture containing 3.00 g (11.98 mmol) of TBBT, 4.71 g (9.58 mmol) of SDCDPS, and 2.65 g (19.17 mmol) of K_2_CO_3_ in 28 mL of *N*-methyl-2-pyrrolidone (NMP) was added into the flask. Afterwards, 14 mL of toluene (NMP/toluene = 2/1, *v*/*v*) was added as an azeotropic agent. To ensure complete dehydration, the reaction mixture was heated to 145 °C for 4 h. After toluene was completely removed, the temperature was increased to 160 °C, and the reaction was continued for another 4 h. After the reaction, the solution was cooled to room temperature and then filtered to remove the salt produced during the polymerization. The homogeneous solution was then poured into IPA to precipitate the polymer, which was filtered, rinsed several times with methanol and IPA alcohol, and dried in a vacuum oven for 12 h at 60 °C. The product polymer yield was 80%.

Thiophenoxide-terminated poly(arylene thioether sulfone) (DT) was also prepared through a condensation polymerization reaction using 3.00 g (11.98 mmol) of TBBT and 2.75 g (9.58 mmol) of DCDPS. Unlike in the preparation of SDT, the reaction mixture for DT synthesis was not subjected to a dehydration process using toluene due to the high reactivity of DT and DCDPS. The homogeneous solution obtained after a reaction at 160 °C for 4 h was poured into methanol and then rinsed with DI water several times. After drying at 60 °C in a vacuum oven for 12 h, DT was obtained at a yield of 91%.

### 2.5. Preparation of Cross-Linked SPAES Membrane

The cross-linked SPAES membranes were prepared through the in-situ casting and heating of SPAES-Cl solutions with different cross-linkers, such as SDT and DT. The cross-linked membranes containing SDT and DT were designated as SDT-CSPAES and DT-CSPAES, respectively. SDT-CSPAES membrane was prepared by dissolving 0.6000 g (1.1261 mmol of repeat units) of SPAES-Cl, 0.0716 g (0.04447 mmol) of SDT, and 0.1160 g (1.1261 mmol) of triethylamine (TEA) in 4.400 mL DMSO. An appropriate amount of SDT was added to ensure that the molar ratio between the potassium thiophenoxide end-group of SDT and the chloromethyl groups of SPAES-Cl was 1:1. The amount of TEA was determined based on the molar content of the potassium thiophenoxide end-group of SDT. Lastly, the amount of DMAc was adjusted to obtain an approximate total concentration of SPAES-Cl, SDT, and TEA of 12 wt%. The solution mixture was spread onto a clean glass plate. The thickness of the film was controlled using a doctor blade. The cast solution was heated to 120 °C in a vacuum oven for 12 h. During the heat treatment, the cross-linked membrane was formed through solvent evaporation and a simultaneous nucleophilic substitution reaction between the chloromethyl groups and potassium thiophenoxide groups. To detach the synthesized film and remove the remaining solvents and TEA, the coated glass plate was soaked in and washed with DI water several times. DT-CSPAES membrane was fabricated through a similar preparation method; however, for this case, DT and DMAc were used as cross-linker and solvent, respectively. After drying in a vacuum oven at 60 °C for 24 h, cross-linked membranes with an approximate thickness of 15–20 µm were obtained.

For comparison, a linear SPAES membrane was prepared by casting a 12 wt% SPAES solution in DMAc onto clean glass. The employed film preparation method, which included solution casting, heat treatment, and drying, was similar to that used to prepare the cross-linked SPAES membranes. The thickness of the synthesized linear SPAES membrane was approximately 25 µm.

### 2.6. Preparation of Membrane Electrode Assemblies (MEAs)

MEAs made from SDT-CSPAES membrane and recast-Nafion were prepared through a typical decal method [25,26]. Catalyst inks were prepared by adding a Pt/C catalyst powder (50 wt%, Tanaka Kikinzoku Kogyo, Tokyo, Japan) into a mixture containing an Aquivion^TM^ ionomer dispersion (EW750, Solvay, Brussels, Belgium) and a water–n-propanol solvent mixture. To ensure the uniformity of the catalyst ink, the dispersion was mixed using an ultrasonic vibrator (Sonic, Oklahoma City, OK, USA, Vibra-cell). Then, a catalyst layer was developed by coating the ink mixture onto a PTFE sheet, which was subsequently dried at 60 °C. The MEA was fabricated by hot pressing the catalyst layer and the membrane together, and then transferring the catalyst layer onto the membrane at 120 °C. On one hand, the Pt loading at the MEA was 0.4 mg/cm^2.^ On the other hand, the ionomer content in the catalyst layer was approximately 30%.

### 2.7. Characterization

^1^H-NMR spectra were recorded using an Avance-400 spectrometer (Bruker, Bremen, Germany) with a proton frequency of 400 MHz. Deuterated dimethylsulfoxide and tetramethylsilane were used as the solvent and internal standard, respectively. The Fourier-transform infrared (FT-IR) spectra of the films were recorded using a Cary 660 FT-IR spectrometer (Agilent Technology, Palo Alto, CA, USA) in the attenuated total reflectance mode at ambient temperature. Data were collected from 32 scans at a 4 cm^−1^ resolution. To avoid differences caused by the pressure and penetration depth, the sample was placed in equal physical contact with the sampling plate of the spectrometer accessory.

For the full cell testing, cells were assembled by placing two gas diffusion layers (TGP-H-060, Toray, Tokyo, Japan) on both sides of the MEA. The performance of each cell (active area = 10 cm^2^) was measured using a fuel cell test station (Scribner Associates Inc., Southern Pines, NC, USA, 850e Fuel Cell Test Station) at ambient pressure and 120 °C. The flow rates of H_2_ at the anode and air at the cathode were 50 and 200 cm^3^ min^−1^, respectively. The inlet gases were humidified to 40% relative humidity (RH). Voltage values at each current density were recorded after 1 min of current application [25,26].

The detailed experimental method for the characterizations of the membranes, including inherent viscosity, solubility test, mechanical properties, field emission scanning electron microscope (FE-SEM), water uptake, area-based swelling ratio, and proton conductivity, are described in the Supplementary Materials.

## 3. Results and Discussion

### 3.1. Synthesis and Modification of SPAES and Polymeric Cross-Linker

**Figure 1:**
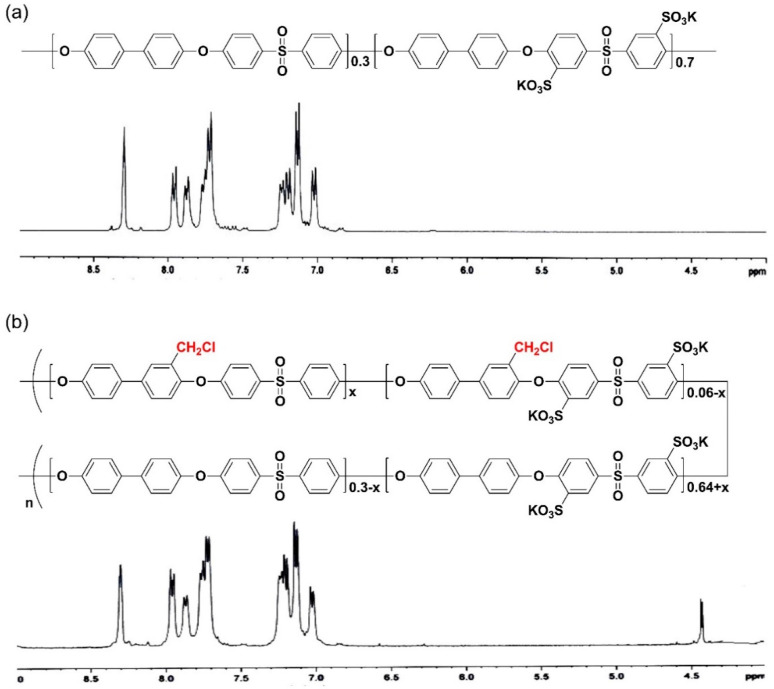
Chemical structures and ^1^H-NMR spectra of (**a**) SPAES and (**b**) SPAES-Cl.

SPAES was synthesized through condensation polymerization. Figure 1 shows the chemical structure and ^1^H-NMR spectra of SPAES and SPAES-Cl. From the ^1^H-NMR analysis, the DS of SPAES (66 mol%) was smaller than the expected value, possibly due to the reactivity of the sulfonated dichloro monomer being lower than that of the unsulfonated dichloro monomer [27]. To introduce cross-linking junction points, chloromethyl groups were introduced into the SPAES backbone. When larger amounts of chloromethyl groups were attached to the SPAES, the polymer solution became too viscous, making it difficult to ensure reproducibility. Therefore, the reaction conditions, such as temperature and time, were controlled to obtain a polymer with a relatively low degree of chloromethylation (approximately 6 mol%). After the modification of SPAES, its inherent viscosity slightly increased, whereas its DS remained the same (Table 1).

**Figure 2:**
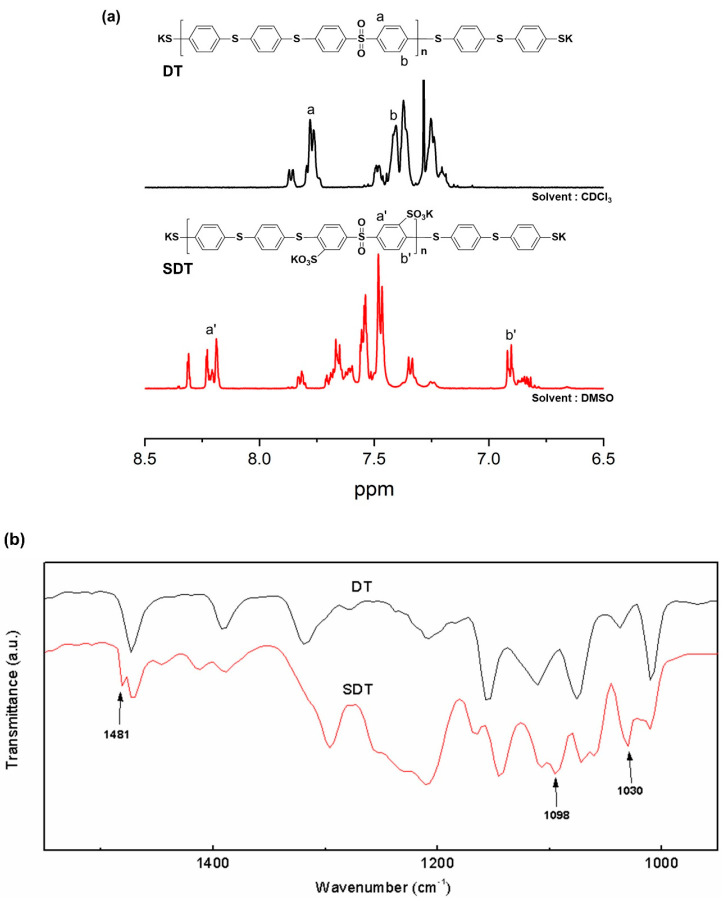
(**a**) ^1^H-NMR and (**b**) FT-IR spectra of DT and SDT.

Two polymers with thiophenoxide-terminated structures (SDT and DT) were synthesized as polymeric cross-linkers (Figure S2). Given their low inherent viscosities (Table 1), the molecular weights of SDT and DT are lower than those of SPAES. This is because the stoichiometry of TBBT was intentionally exceeded to obtain a thiol-end-capped polymer for cross-linking. The polymer with the sulfonated groups was a hydrophilic crosslinker, while the one without the sulfonated groups was a hydrophobic cross-linker. The different hydrophilicities of the polymeric cross-linkers are expected to have different effects on the membrane properties. To confirm the chemical structure of SDT and DT, ^1^H-NMR and FT-IR analyses were carried out (Figure 2a,b). The only difference between the two polymers is the presence (or absence) of the sulfonated groups due to their similar chemical structures. Characteristic peaks for the tri-substitution on an aromatic ring and the symmetric and asymmetric stretching vibrations of sulfonated groups were observed in the FT-IR spectrum of SDT at 1481, 1030 and 1098 cm^−1^, respectively [28,29,30].

### 3.2. Preparation of Cross-Linked SPAES Membranes

**Figure 3:**
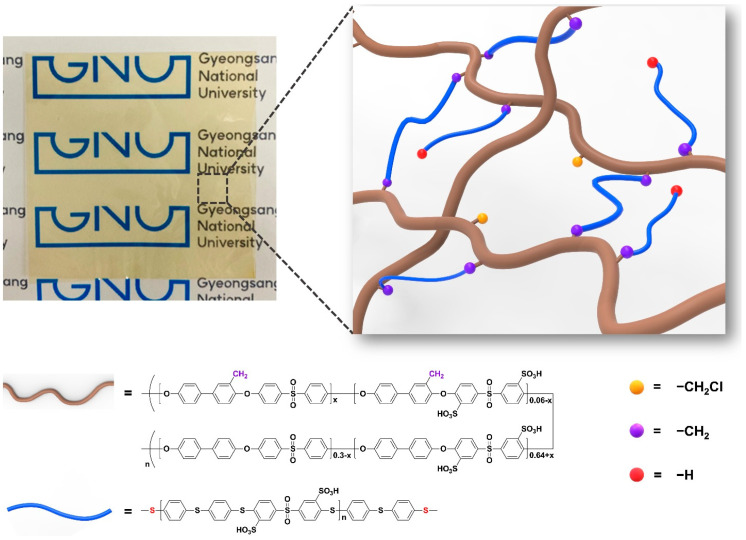
Schematic illustration of cross-linked SPAES membranes prepared with hydrophilic polymeric cross-linker (SDT).

Transparent and flexible cross-linked SPAES membranes were prepared through the in situ casting and heating of a DMAc solution containing SPAES-Cl and cross-linkers (SDT or DT) with a small amount of a liquid base catalyst (TEA) (Figure 3). The cross-linked structure can be formed through a nucleophilic substitution reaction between the chloromethyl groups at the SPAES backbone and potassium thiophenoxide groups at the end of the cross-linkers (SDT or DT), alongside solvent evaporation during heat treatment. Figure S3 shows the possible chemical structure of SDT-CSPAES membranes. The success of the formation of the cross-linked structures in the membranes and their cross-linking densities were evaluated through solubility and gel fraction tests. Table 2 summarizes the solubility test results for all membranes. On one hand, the linear SPAES membrane dissolved and became largely swollen in most organic solvents owing to its high DS [11,31]. On the other hand, none of the cross-linked membranes dissolved in the organic solvents. Furthermore, they became only slightly swollen in polar solvents. In addition, DT-CSPAES membranes had a lower tendency to swell than did SDT-CSPAES membranes, which can be attributed to the different hydrophilicities of the polymeric cross-linkers (SDT and DT). The gel fraction can be used to estimate the ratio of the cross-linked portion in the membrane [32]. As shown in Figure S4, the linear SPAES membrane totally dissolved in DMAc. In contrast, the cross-linked membranes retained their shape with a gel fraction value larger than 60 wt%. However, slight swelling was observed for the SDT-CSPAES membrane. The higher gel fraction of DT-CSPAES membrane (68.1%) than that of SDT-CSPAES membrane (62.5%) indicates that DT, which is less hydrophilic than SDT, was more effective in forming the stable cross-linked structure. These results indicate that the cross-linked structure was successfully induced using the polymeric cross-linkers. From the cross-sectional SEM images in Figure S5, the thickness of SDT-CSPAES and DT-CSPAES membranes were 20 and 19 µm, respectively.

### 3.3. Swelling Ratio, Water Uptake, and Mechanical Properties of the Cross-Linked Membranes

**Figure 4:**
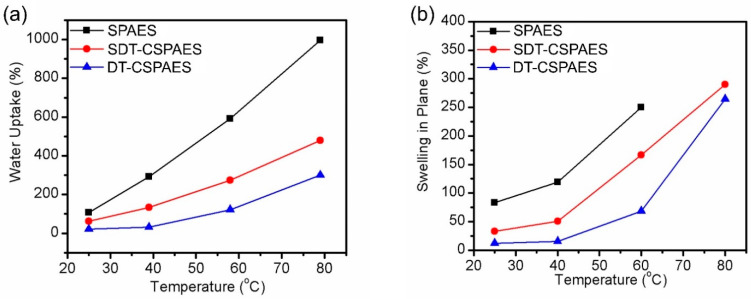
(**a**) Water uptake and (**b**) area-based swelling ratio of all the membranes.

To assess membrane stability, the water-absorbing ability and swelling behavior of the cross-linked membranes were compared to those of the linear SPAES membrane. Figure 4 shows the water uptake values and swelling ratios of the membranes. As the temperature was increased, both the water uptake and swelling ratio of the membranes increased rapidly. The swelling ratio of the linear SPAES membrane beyond 60 °C could not be measured because of its excessive swelling at these temperatures. In contrast, the water uptake values and swelling ratios of the cross-linked membranes were much lower than those of the linear SPAES membrane because the cross-linked network prevents excessive swelling by water absorption [33,34]. Therefore, the cross-linked structure produced using polymeric cross-linkers was effective in enhancing the dimensional stability of the membranes. Meanwhile, the cross-linked membranes produced in this work exhibited relatively higher water uptake and swelling ratios than those of previously reported cross-linked membranes with similar DS values [11,24,27]. This is possibly due to the flexibility and long length of the polymeric cross-linkers with a sulfide bond structure. After cross-linking, many free volumes among the polymer chains remain. This allows more water to be absorbed in the membranes, which ultimately increases their swelling ratio. In addition, the SDT-CSPAES membrane registered a relatively higher water uptake and swelling ratio than the DT-CSPAES membrane due to its hydrophilic SDT cross-linkers, which can attract more water than DT. Oxidative stability of the membranes was investigated by the Fenton’s test (Table S1). As expected, both cross-linked membranes are much more stable than the linear SPAES membrane.

**Figure 5:**
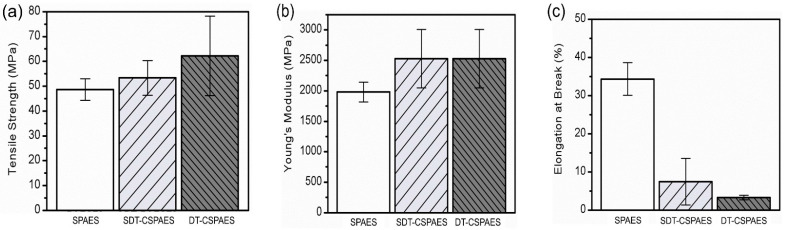
(**a**) Tensile strength, (**b**) Young’s modulus, and (**c**) elongation at break values of all the membranes at 25 °C and 40% RH.

Figure 5 shows the mechanical properties, such as tensile strength, Young’s modulus, and elongation at break, of the membranes. The tensile strength and elongation at break of the linear SPAES membrane were 47 MPa and 34%, respectively. In comparison, the cross-linked membranes exhibited higher tensile strength and Young’s modulus and lower elongation at break than linear SPAES membrane, because the cross-linked structure strengthens the mechanical toughness and lowers the polymer chain mobility [34,35]. Comparing the effects of two cross-linkers, DT was found to be a more effective cross-linker in increasing the mechanical strength of linear SPAES because of the high cross-linking density of the DT-CSPAES membrane assumed from the gel fraction value. However, the brittleness of this membrane, which can be seen from its very low elongation at break value (4%), makes it difficult to use it as a membrane for practical fuel cell operations. Therefore, considering its improved tensile strength and Young’s modulus, and reasonable elongation at break, the SDT-CSPAES membrane is deemed more appropriate for practical applications.

### 3.4. Proton Conductivity and Cell Performance

**Figure 6:**
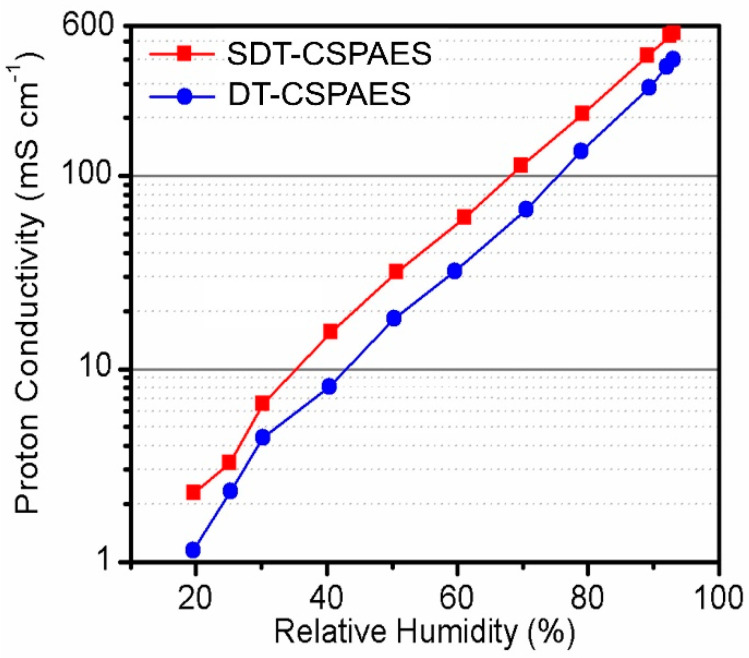
Proton conductivity of SDT-CSPAES and DT-CSPAES membranes at 120 °C as a function of RH.

Figure 6 shows the proton conductivities of the cross-linked membranes measured at 120 °C and 20–95% RH. On one hand, the conductivity of the linear SPAES membrane cannot be obtained under the measurement conditions because of its poor stability originating from its high DS (70 mol%) [25]. On the other hand, SDT-CSPAES and DT-CSPAES membranes exhibited high proton conductivities due to their improved stability after the introduction of the cross-linked structures. Regardless of the RH setting, the proton conductivity of SDT-CSPAES membrane was higher than that of DT-CSPAES. Case in point, at 40% RH, the proton conductivity of SDT-CSPAES and DT-CSPAES membranes were 15.48 and 8.07 mS cm^−2^, respectively. Similar to previous findings, the high proton conductivity of SDT-CSPAES membranes can be ascribed to the high hydrophilicity of SDT, which allowed the SDT-CSPAES membranes to absorb water more efficiently than the DT-CSPAES membranes. Since SDT has two sulfonic acid groups per repeat unit, the number of sulfonic acid groups in the membrane after cross-linking would increase. This results in a relatively larger water uptake and forms large hydrophilic domains that may facilitate proton transfer, resulting in high proton conductivity even under very low RH conditions [36]. As a result, SDT-CSPAES membranes exhibited high proton conductivity despite the inevitable reduction in the chain mobility by the formation of the cross-linked structure. In contrast, the proton conductivity of the DT-CSPAES membrane decreased considerably due to the absence of sulfonic acid groups in DT, which would decrease the total sulfonic acid group density in the membrane after cross-linking. The different ion-exchange capacity (mequiv. g^−1^) between the SDT-CSPAES and DT-CSPAES membranes, indicating the presence of different numbers of ion exchangeable groups per unit mass of the dry membrane, also supported the conductivity difference of these two membranes.

**Figure 7:**
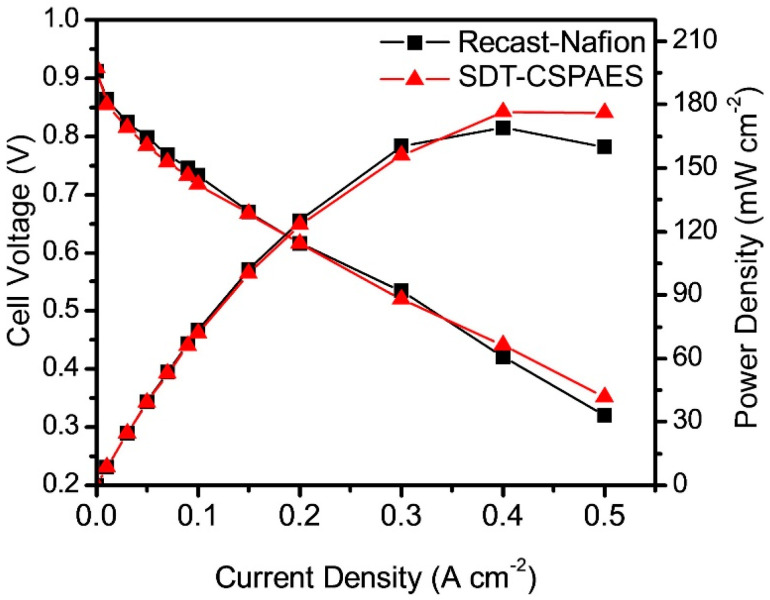
Cell performance of the MEAs made from recast-Nafion and SDT-CSPAES membrane at 120 °C under 40% RH with H_2_/air condition.

Based on the measured mechanical properties and proton conductivity, the SDT-CSPAES membrane was used for the fuel cell performance evaluation. Since the cell performance of linear SPAES membrane cannot be measured due to the failure of the membrane in the activation step, recast-Nafion (20–25 μm) was used as a reference membrane for comparison. Figure 7 shows the polarization curves at 120 °C and 40% RH of the MEAs made from the SDT-CSPAES membrane and recast-Nafion. The MEA made from SDT-CSPAES membrane registered a slightly higher maximum power density (176.4 mW cm^−2^) than that made from the recast-Nafion (169.0 mW cm^−2^). The expected cell performance improvement corresponding to the high proton conductivity of the SDT-CSPAES membrane was not achieved because the MEA preparation processes and components were optimized for the PFSA ionomer membranes. Nevertheless, the results demonstrate that the SDT-CSPAES membrane prepared with use of a highly sulfonated polymer and hydrophilic polymeric cross-linker was an effective cross-linked membrane for PEMFC applications.

## 4. Conclusions

In this study, cross-linked SPAES membranes were synthesized using polymeric cross-linkers through a facile in situ casting method. With the aid of polymeric cross-linkers, the cross-linked membranes exhibited much improved physicochemical properties, including dimensional stability and mechanical strength, given the high degree of sulfonation (DS) value of SPAES. Additionally, the effects of the hydrophilicity of the cross-linkers on the stability and proton conductivity of the cross-linked membranes were studied. The water absorption ability of the cross-linked membrane prepared using the hydrophilic SDT cross-linkers increased without a considerable reduction in membrane stability; thus, the membrane exhibited relatively high proton conductivity at high temperature and low RH. As a result, the cell performance of the MEA made from this membrane is even higher with the maximum power density of 176.4 mW cm^−2^ than that of the MEA made from recast-Nafion. Therefore, we concluded that the cross-linked membranes made from highly hydrophilic polymeric cross-linkers via a facile casting process are applicable to various PEM materials prepared using highly sulfonated polymers for practical PEMFC applications. In addition, since the construction of the cross-linked structure of the CSPAES membrane system is easily tuned by changing the type of polymeric cross-linkers and the modification degree of the SPAES main chain, this cross-linking strategy could apply in various polymer electrolytes used in energy devices.

## Figures and Tables

**Table 1:** Degree of sulfonation values and inherent viscosity of SPAES, SPAES-Cl, SDT and DT.

Polymer	Structure	Degree of Sulfonation (%)		*η*_inch_^c^ (dL/g)
		Monomer ^a^	^1^H-NMR ^b^	
SPAES	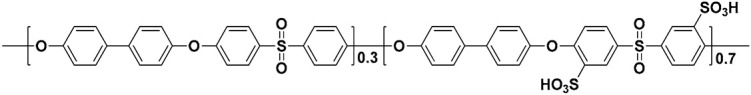	70	66	1.91
SPAES-Cl	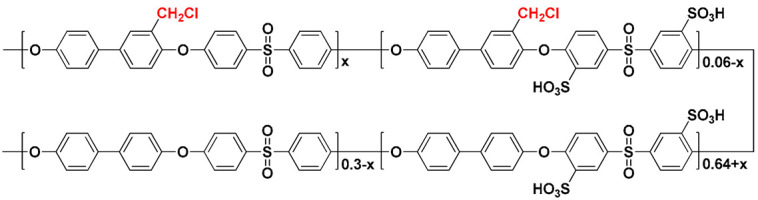	70	66	1.95
SDT	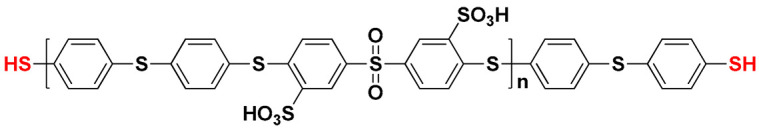	100	>95	0.93
DT	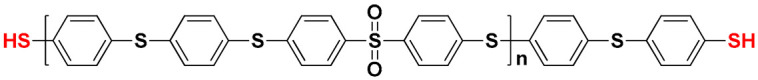	0	0	1.06

^a^ Molar ratio of sulfonated dichloro monomer to unsulfonated dichloro monomer. ^b^ Calculated by comparison of the peaks from ^1^H-NMR spectra. ^c^ Measured at 25 °C.

**Table 2:** Solubility test of SPAES, SDT-CSPAES and DT-CSPAES membranes.

Polymer	Solvents						
	DMAc	NMP	DMSO	Methanol	Ethanol	THF	Acetone
SPAES	S ^a^	S	S	Sw	Sw	Sw	Sw
SDT-CSPAES	Sw ^b^	Sw	Sw	Sw	Sw	I	I
DT-CSPAES	Sw	Sw	Sw	I ^c^	I	I	I

^a^ Soluble at room temperature. ^b^ Swelled at room temperature. ^c^ Insoluble at room temperature.

## Data Availability

Not applicable.

## Supplementary Materials

The following supporting information can be downloaded at: https://www.mdpi.com/article/10.3390/membranes13010007/s1, Figure S1. Synthetic scheme of sulfonated poly(arylene ether sulfone) (SPAES) with degree of sulfonation of 70 mol% and SPAES with chloromethyl moieties (SPAES-Cl); Figure S2. Synthetic scheme of (a) thiophenoxide-terminated sulfonate poly(arylene thioether sulfone) (SDT) and (b) thiophenoxide-terminated poly(arylene thioether sulfone) (DT); Figure S3. Chemical structure of cross-linked membrane prepared with hydrophilic cross-linker (SDT-CSPAES); Figure S4. Gel fraction test of all the membranes using DMAc at 60 °C for 6 h; Figure S5. Cross sectional SEM images (×1000) of (a) SDT-CSPAES and (b) DT-CSPAES membranes; Table S1. Oxidative stability and ion exchange capacity of membranes [37].
