# Supplementary material for: Cross-Linked Sulfonated Poly(arylene ether sulfone) Membrane Using Polymeric Cross-Linkers for Polymer Electrolyte Membrane Fuel Cell Applications
Source: Membranes (Basel). 2022 Dec 21;13(1):7. doi: 10.3390/membranes13010007 (PMC9861409; doi:10.3390/membranes13010007)
Supplement: Supplementary file 1 [file membranes-13-00007-s001.zip › membranes-2110388-supplementary.pdf]

Supporting Information of

Cross-linked sulfonated poly(arylene ether sulfone) membrane using polymeric cross-linkers for polymer electrolyte membrane fuel cell applications

Junghwan Kim[1], Seansoo Hwang[2], Yu-Gyeong Jeong[2], Yong-Seok Choi[3,*] and Kihyun Kim[2,*]

[1] Center for Hydrogen·Fuel Cell Research, Korea Institute of Science and Technology (KIST), Hwarang-ro 14-gil 5, Seongbuk-gu, Seoul, 02792, Republic of Korea

[2] Department of Materials Engineering and Convergence Technology, Gyeongsang National University, Jinju 52828, Republic of Korea

[3] Composites Materials Application Research Center, Korea Institute of Science and Technology, 92 Chudong-ro, Bongdong-eup, Wanju-gun, Jeonbuk 55324, Republic of Korea

* Correspondence: choiys@kist.re.kr (Y.-S.C.); kihyun@gnu.ac.kr (K.K.); Tel.: +82-63-219-8152 (Y.-S.C.); +82-55-772-1655 (K.K.)

Materials

4,4'-dichlorodiphenyl sulfone (DCDPS, 98%, Aldrich) and 4,4'-dihydroxybiphenyl (BP, 97.0%, Aldrich) were purified through recrystallization in toluene and methanol, respectively. 3,3'-Disulfonated-4,4'-dichlorodiphenylsulfone (SDCDPS) was prepared from DCDPS according to a procedure in a previous report [1]. After recrystallization from a mixture of isopropyl alcohol (IPA) and deionized (DI) water (7/3 v/v ratio), the SDCDPS yield was 85%. Prior to use, 4,4'-Thiobisbenzenethiol (TBBT, 98%, Aldrich) and K_2CO_3 (99.0%, Aldrich) were vacuum dried for 48 h. $SnCl_4$ (99%, Aldrich), chloromethyl methyl ether (95.0%, Kanto Chemical), and triethylamine (TEA, 99.0%, TCI) were used as received. *N,N*-Dimethylacetamide (DMAc, 99%, Junsei), *N*-Methyl-2-pyrrolidone (NMP, 99.0%, Junsei), and dimethyl sulfoxide (DMSO, 99%, Junsei) were stored over molecular sieves under N_2 gas environment. Toluene (99.5%, Junsei) was refluxed over calcium hydride and then distilled. All other solvents and reagents were used as received.

Characterization

The inherent viscosity was measured using an Ubbelode viscometer in a water bath at 30 °C. The sample used for the inherent viscosity measurements was a solution containing dried polymer powder (70 °C for 1 h) in DMAc (concentration = 0.3 g dL^{-1}).

Solubility tests were performed by immersing the membranes in different solvents at 30 and 80 °C for 1 h. Gel fraction tests were performed through a well-known solvent-extraction method. The dry membranes (2 cm × 2 cm) were weighed (W_1) and refluxed in excess DMAc at 80 °C for 12 h. Afterwards, the membranes were washed for several times with DI water and dried at 80 °C in a vacuum oven until a constant weight (W_2) was obtained. Gel fractions were

calculated using Equation (1).

Gel fraction [%] = $W_2/W_1 \times 100$ (S1)

The water uptake and area-based swelling ratio of the membranes were calculated by measuring the differences in the weight and area of the dry and swollen membranes. The dry membranes were cut into 1 cm × 5 cm rectangles. Their weights and volumes were obtained. Then, the membranes were immersed in DI water for 3 h at temperatures ranging from 25 to 80 °C. The weights were measured after the membranes were removed and wiped with tissue paper. The water uptake and area-based swelling ratio of the membranes were calculated using the following equations,

Water uptake [%] = $[(W_{wet}-W_{dry})/W_{dry}] \times 100$ (S2)

Area-based swelling ratio [%] = $[(1+\Delta L)(1+\Delta W)-1] \times 100$, (S3)

where W_{dry} and W_{wet} are the weights of the dry and wet membranes, respectively. ΔL and ΔW represent the changes in the length and width of the membrane, respectively. The oxidative stability of the membranes was evaluated by Fenton's test by observing the dissolution behavior of the samples after being exposed to a Fenton's reagent (3 wt.% H_2O_2 aqueous solution containing 16 ppm Fe^{2+}). After immersing the membranes in the Fenton's reagent at 70 °C, the membranes were observed periodically and the times when the membranes began to break into small pieces (τ_1) and completely dissolved (τ_2) were recorded, respectively.

The mechanical properties of the membranes were evaluated using a universal testing machine (Lloyd-LS1, UK). ASTM standard D638 (Type V specimens) was used for the preparation of the dumbbell-shaped specimens. The measurements were performed at 25 °C

and 40% relative humidity (RH) with gauge length and crosshead speed of 15 mm and 5 mm min^{-1}, respectively. Each measurement was composed of at least five sample readings; the average value of these five readings was calculated. Field emission scanning electron microscope (FE-SEM) images were obtained by using a JSM-6700F (JEOL, Japan). Cryo-fractured membranes prepared in liquid nitrogen was used to investigate the cross-sectional morphology.

The proton conductivities of the membranes were measured at 120 °C under various RH conditions through the BekkTech Test Protocol. The samples were fitted to a four-point measurement cell and connected to a test station (BekkTech BT-552MX) for simultaneous RH and temperature control. The temperatures of the cell and humidification column were controlled to ensure that the desired RH was achieved. The membranes were pre-equilibrated at 120 °C and 70% RH for 2 h. Initially, the tests were carried out at 120 °C of H_2 and 230 kPa under an input flow rate of 500 cm^3 min^{-1}. Afterwards, conductivity measurements were performed under decreasing (70% to 20% RH) and then increasing (90% RH) humidity. The RH setting was modified at 10% intervals with a 15 min equilibration time for each step. Ion-exchange capacity (IEC) of the membranes was determined by typical back-titration method. The samples were soaked in 1 M NaCl aqueous solution for 4 h, and then the solutions were titrated with 0.01 M NaOH aqueous solution. The IEC value was calculated using the following equation:

$$\mathrm{IEC}\ [\mathrm{mequiv.g^{-1}}] = (C_{NaOH} \cdot \Delta V_{NaOH} / W_s) \times 1000 \qquad \text{(S4)}$$

where C_{NaOH}, ΔV_{NaOH}, and W_s are the concentration of NaOH (aq), the consumed volume of NaOH (aq), and the weight of the dry membrane, respectively.

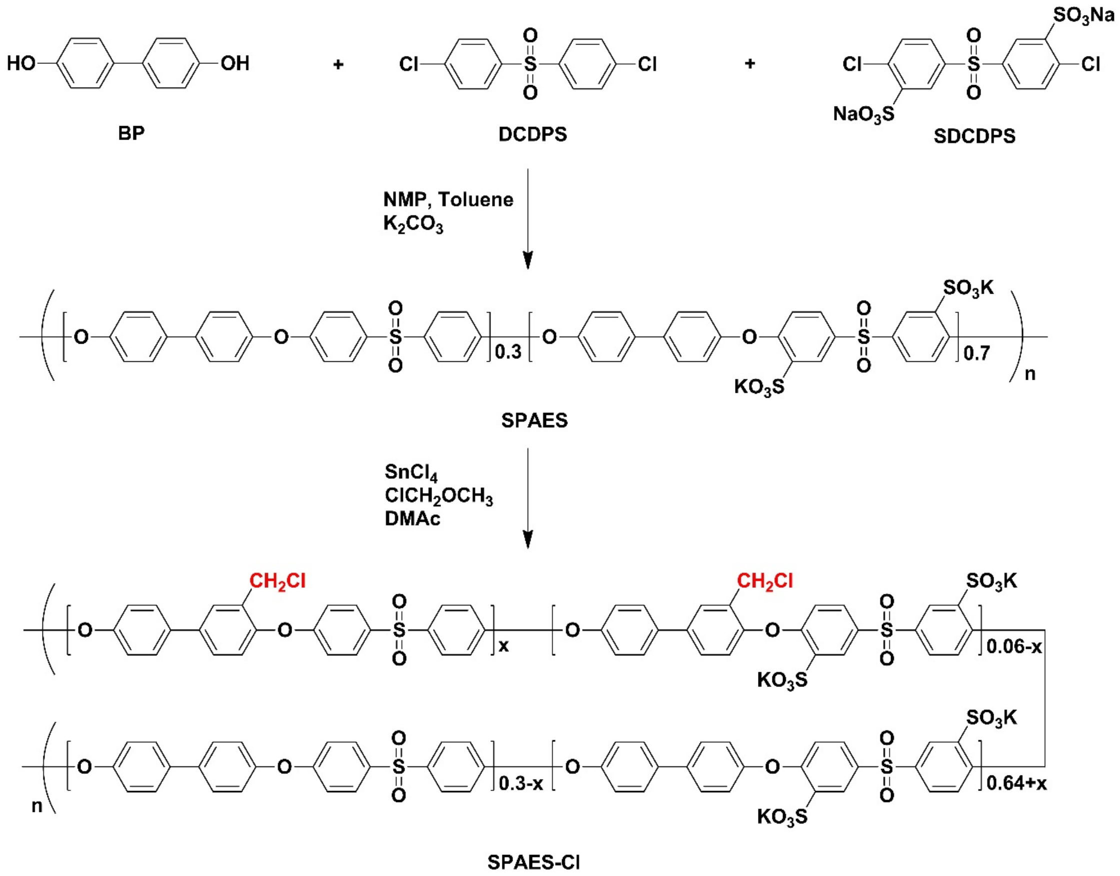

Figure S1. Synthetic scheme of sulfonated poly(arylene ether sulfone) (SPAES) with degree of sulfonation of 70 mol% and SPAES with chloromethyl moieties (SPAES-Cl).

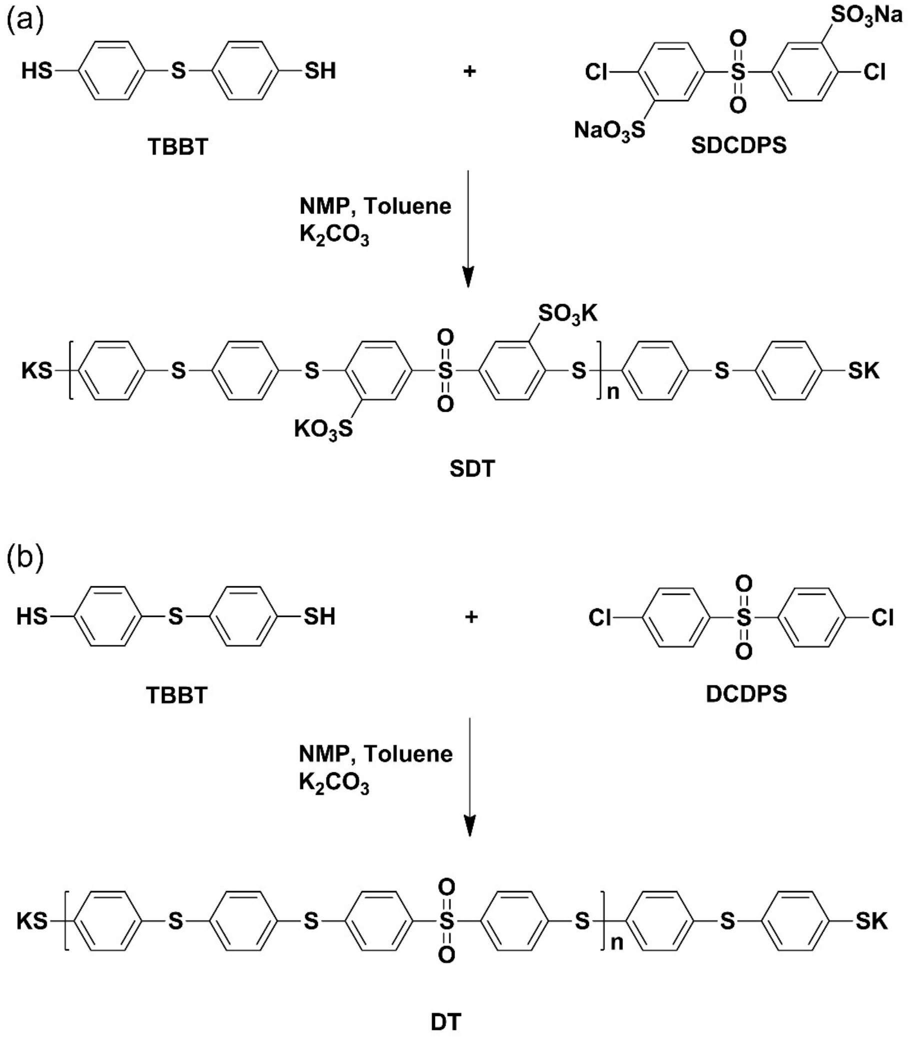

Figure S2. Synthetic scheme of (a) thiophenoxide-terminated sulfonate poly(arylene thioether sulfone) (SDT) and (b) thiophenoxide-terminated poly(arylene thioether sulfone) (DT).

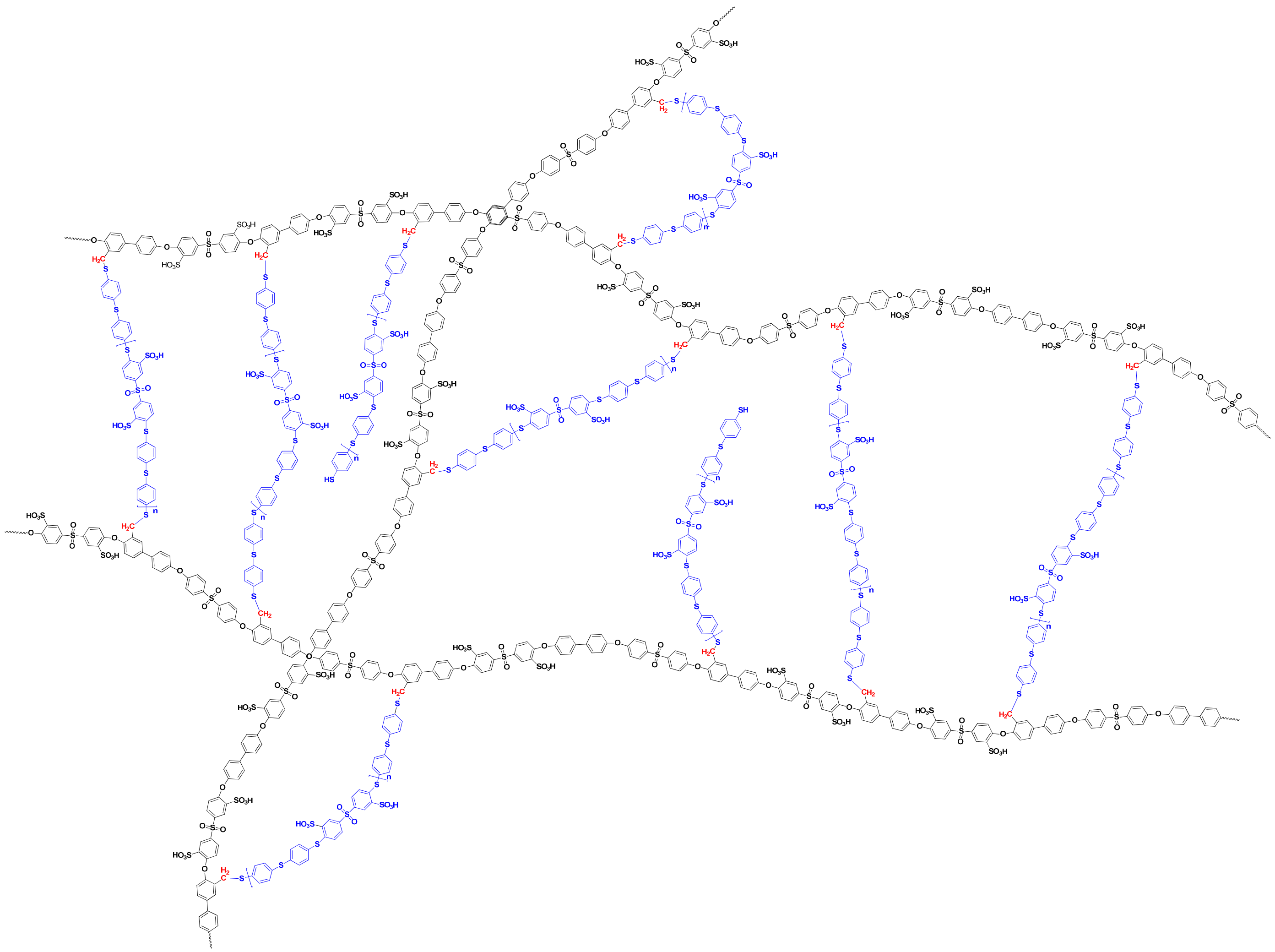

Figure S3. Chemical structure of cross-linked membrane prepared with hydrophilic cross-linker (SDT-CSPAES).

Polymer	Gel fraction [a]	
	State	wt. %
SPAES	S [b]	66
SDT-CSPAES	Sw [c]	62.5
DT-CSPAES	Sw [c]	68.1

[a] Measured after immersed in DMAc (60 °C, 6 h).
[b] Soluble at room temperature.
[c] Swelling at room temperature.

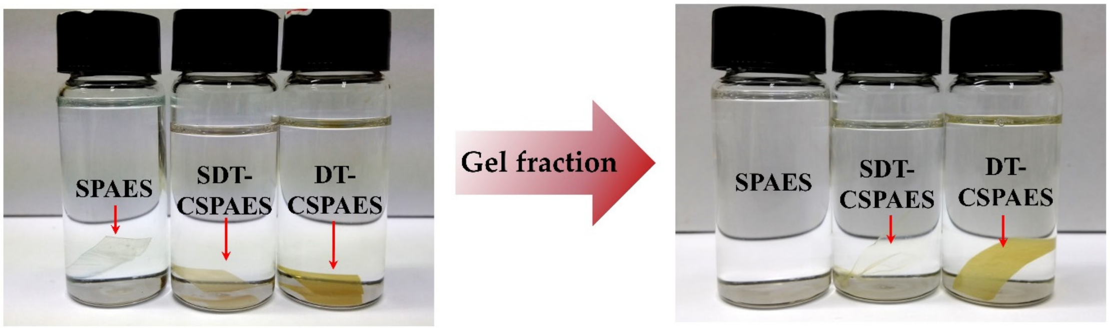

Figure S4. Gel fraction test of all the membranes using DMAc at 60 °C for 6 h.

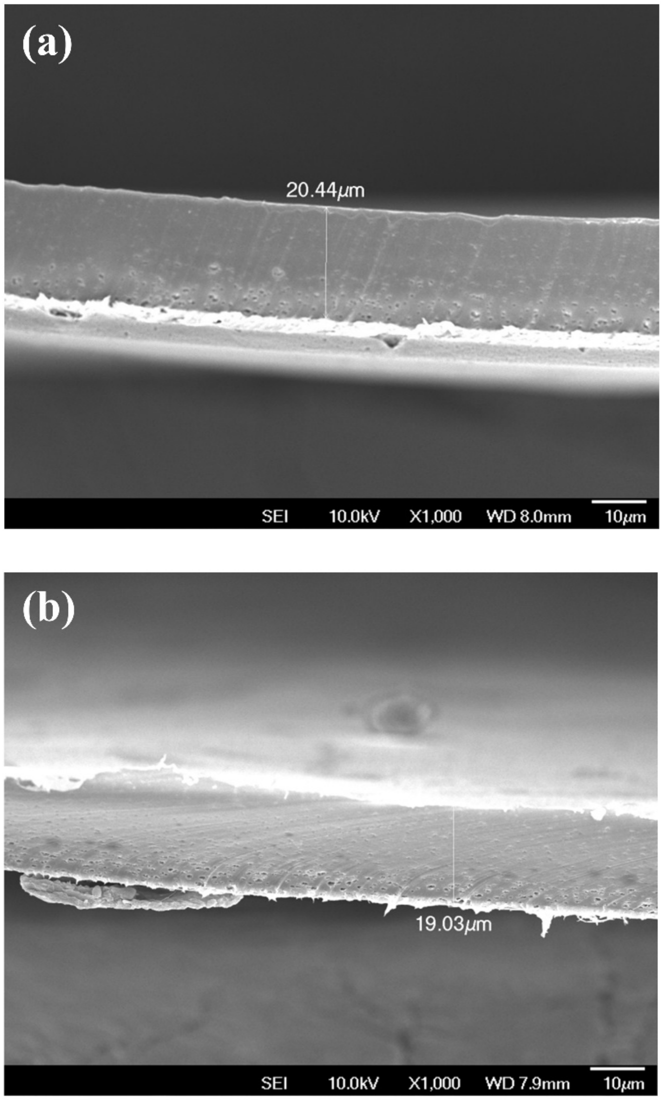

Figure S5. Cross sectional SEM images (×1000) of (a) SDT-CSPAES and (b) DT-CSPAES membranes.

Table S1. Oxidative stability and ion exchange capacity of membranes.

Membrane	Fenton's test		IEC [c]
	τ_1 [a] (min)	τ_2 [b] (min)	(mequiv. g^{-1})
SPAES	80	100	2.52
SDT-CSPAES	150	180	2.38
DT-CSPAES	190	250	2.12

[a] The time when the membranes began to break into pieces in Fenton's reagent at 70 °C.
[b] The time when the membranes dissolved completely in Fenton's reagent at 70 °C.
[c] Measured by back titration method.

References

[1] Ueda, M.; Toyota, H.; Ouchi, T.; Sugiyama, J.I.; Yonetake, K.; Masuko, T.; Teramoto, T. Synthesis and characterization of aromatic poly (ether sulfone) s containing pendant sodium sulfonate groups. *J. Polym. Sci., Part A: Polym. Chem*. **1993**, *31*, 853-858.
